# Comparative secretome analysis of different smut fungi and identification of plant cell death-inducing secreted proteins from *Tilletia horrida*

**DOI:** 10.1186/s12870-019-1924-6

**Published:** 2019-08-16

**Authors:** Aijun Wang, Linxiu Pan, Xianyu Niu, Xinyue Shu, Xiaoqun Yi, Naoki Yamamoto, Shuangcheng Li, Qiming Deng, Jun Zhu, Yueyang Liang, Lingxia Wang, Ping Li, Aiping Zheng

**Affiliations:** 1 Rice Research Institute of Sichuan Agricultural University, Chengdu, China; 2 Key laboratory of Sichuan Crop Major Disease, Sichuan Agricultural University, Chengdu, China; 3 Key Laboratory of Southwest Crop Gene Resource and Genetic Improvement of Ministry of Education, Sichuan Agricultural University, Yaan, China

**Keywords:** *Tilletia horrida*, Effector proteins, Cell-death, Signal peptides, RNase active site, Yeast two-hybrid

## Abstract

**Background:**

*Tilletia horrida* is a basidiomycete fungus that causes rice kernel smut, one of the most important rice diseases in hybrid rice growing areas worldwide. However, little is known about its mechanisms of pathogenicity. We previously reported the genome of *T. horrida*, and 597 genes that encoded secreted proteins were annotated. Among these were some important effector genes related to pathogenicity.

**Results:**

A secretome analysis suggested that five Tilletia fungi shared more gene families than were found in other smuts, and there was high conservation between them. Furthermore, we screened 597 secreted proteins from the *T. horrida* genome, some of which induced expression in host-pathogen interaction processes. Through transient expression, we demonstrated that two putative effectors could induce necrosis phenotypes in *Nicotiana benthamiana*. These two encoded genes were up-regulated during early infection, and the encoded proteins were confirmed to be secreted using a yeast secretion system. For the putative effector gene smut_5844, a signal peptide was required to induce non-host cell death, whereas ribonuclease catalytic active sites were required for smut_2965. Moreover, both putative effectors could induce an immune response in *N. benthamiana* leaves. Interestingly, one of the identified potential host interactors of smut_5844 was laccase-10 protein (OsLAC10), which has been predicted to be involved in plant lignification and iron metabolism.

**Conclusions:**

Overall, this study identified two secreted proteins in *T. horrida* that induce cell death or are involved in defense machinery in non-host plants. This research provides a useful foundation for understanding the interaction between rice and *T. horrida*.

**Electronic supplementary material:**

The online version of this article (10.1186/s12870-019-1924-6) contains supplementary material, which is available to authorized users.

## Background

The basidiomycete *Tilletia horrida* is a biotrophic fungal pathogen that causes rice kernel smut (RKS), a disease that is distributed throughout hybrid rice growing areas worldwide [1, 2]. *T. horrida* was first reported in 1896, and infects rice floral organs during the flowering stage [3]. A major feature of this pathogen is that it affects both the yield and quality of hybrid seeds by producing masses of dark powdery teliospores [4]. The incidence of *T. horrida* has been recorded to be as high as 87 and 100% in hybrid rice fields in Pakistan and China, respectively [5]. RKS is now an increasing threat to rice cultivation in Asia, Oceania, Europe, America, and Africa [6, 7].

Biotrophic fungi derive nourishment from host cells and tissues for colonization and growth, therefore, they generally do not prevent plant growth and development. On the other hand, the pathogens can secrete many effectors into host cells that suppress plant immune responses; these may be localized to different cellular compartments where they may assume diverse cellular functions to promote infection [8, 9]. For example, host transcription, chromatin remodeling, and immune responses may be affected by secreted effectors [10].

Although plant pathogenic fungi can secrete a large number of proteins, only a small proportion of these have been characterized as effectors. *Magnaporthe oryzae* was the first plant pathogen fungus whose genome was sequenced; subsequently, several effectors in *M. oryzae* have been reported, including Slp1, MoHEG13, and MoHEG16 [11, 12]. In species of smut fungi, several effectors have been studied, including maize *Ustilago maydis* Pit2, See1, Pep1, Cmu1, and Tin2 [13–17]. Pit2 can inhibit the activity of host cellular proteases, which play an important role in plant immune responses [13]. See1 is a fungal effector that directly and specifically contributes to the formation of leaf tumors in maize [14]. Pep1 has an important role in the process by which *U. maydis* and barley covered smut fungus *Ustilago hordei* penetrates the host, and has a conserved function in establishing host-smut pathogen interaction [15]. In contrast with these models, very little is known about the mechanisms of action of *T. horrida* effectors.

Plant receptor proteins that trigger defense responses can recognize effectors, and the functions of several plant receptor proteins function have been reported. For example, the receptor-like proteins Cf-4, Cf-2, Cf-9, and Cf-4E interact with the effectors Avr4, Avr2, Avr9 and Avr4E, respectively, in the tomato pathogen *Cladosporium fulvum* [18, 19]. Previous reports have shown that non-host recognition of effectors is very important for non-host resistance during attempted inoculation by non-host pathogens [20, 21]. Eleven secreted effectors in *Ustilaginoidea virens* have been noted to induce non-host cell death when transiently expressed in *Nicotiana benthamiana* [22]. Effectors are often recognized in plant defense signaling pathways.

According to genome sequencing, *T. horrida* encodes 597 secreted proteins, of which 131 are predicted to be effectors [23]. Furthermore, many potential effector genes are arranged in clusters and transcriptome analyses during infection suggest that putative secreted effectors have an essential role in establishing successful infection in *T. horrida* [23]. No effector genes of *T. horrida* have been functionally characterized. In this study, using transient expression assays, we identified two putative effectors that could induce cell death in *N. benthamiana*. The predicted signal peptides (SPs) and RNase active sites were shown to function differently in the cell death–inducing activity of two putative effectors.

## Results

### Comparative analysis of *T. horrida* secreted proteins with other smut pathogens

**Fig. 1:**
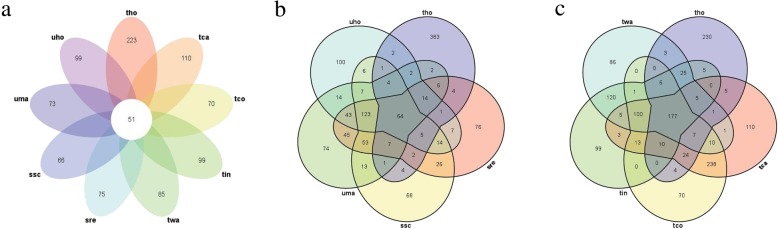
Homology analysis of *Tilletia horrida* secreted proteins with other smut fungi. **a** Venn diagram showing orthologs between the nine smut fungi secreted proteins. **b** Venn diagram showing orthologs between the secreted proteins of the five different smut fungus genera. **c** Venn diagram showing orthologs between the five Tilletia fungi secreted proteins. tca denotes *Tilletia caries*; tco denotes *Tilletia controversa*; tin denotes *Tilletia indica*; twa denotes *Tilletia walkeri*; tho denotes *Tilletia horrida*; sre denotes *Sporisorium reilianum*; ssc denotes *Sporisorium scitamineum*; uma denotes *Ustilago maydis*; uho denotes *Ustilago hordei*

To identify the conservation of secreted proteins between different smut pathogens, the secreted proteins of nine different smut fungi were predicted, including the three smut genera Tilletia, Ustilago, and Sporisorium. The results showed that *T. horrida*, *Tilletia caries*, *Tilletia controversa*, *Tilletia indica*, and *Tilletia walker* possessed more secreted proteins than *Sporisorium scitamineum*, *Sporisorium reilianum*, *U. hordei*, and *U. maydis* (Table 1; Additional file 1: Table S1). We further screened the similarities among the secreted proteins of *T. horrida* and those of eight other smut fungi though an analysis of the gene families of secreted proteins. There were 498 gene families found in 597 secreted proteins of *T. horrida* (Table 1; Fig. 1)*.* Among them, 51 gene families were shared among *T. horrida* and eight other smut fungi; 64 gene families were shared among *T. horrida* and *S. scitamineum*, *S. reilianum*, *U. horde*, and *U. maydis*; 177 gene families were shared among five Tilletia fungi; and 230 gene families appeared to be unique to *T. horrida* (Fig. 1a, b, c). The data showed that *T. horrida*, *T. caries*, *T. controversa*, *T. indica*, and *T. walker* shared more gene families than other smut genera. Further, a high level of conservation among the five Tilletia fungi was observed; this finding is consistent with close relationship between these species.

**Table 1:** The encode gene number of predicted secreted proteins from nine smut fungal isolates

strains	Number of predicted secrete proteins	Number of gene families in secrete proteins	Number of Small predicted secrete proteins (≤400aa)	Small secreted proteins/all secrete proteins (%)
*T. horrida*	597	498	367	61.47
*T. caries*	768	471	516	67.19
*T. controversa*	725	660	479	66.01
*T. indica*	631	569	379	60.06
*T. walker*	600	542	354	59.00
*S. scitamineum*	416	398	230	55.29
*S. reilianum*	505	484	313	61,98
*U. hordei*	430	407	277	64.42
*U. maydis*	509	471	311	61.10

### Genome mining for candidate *T. horrida* small secreted protein encoding genes

**Fig. 2:**
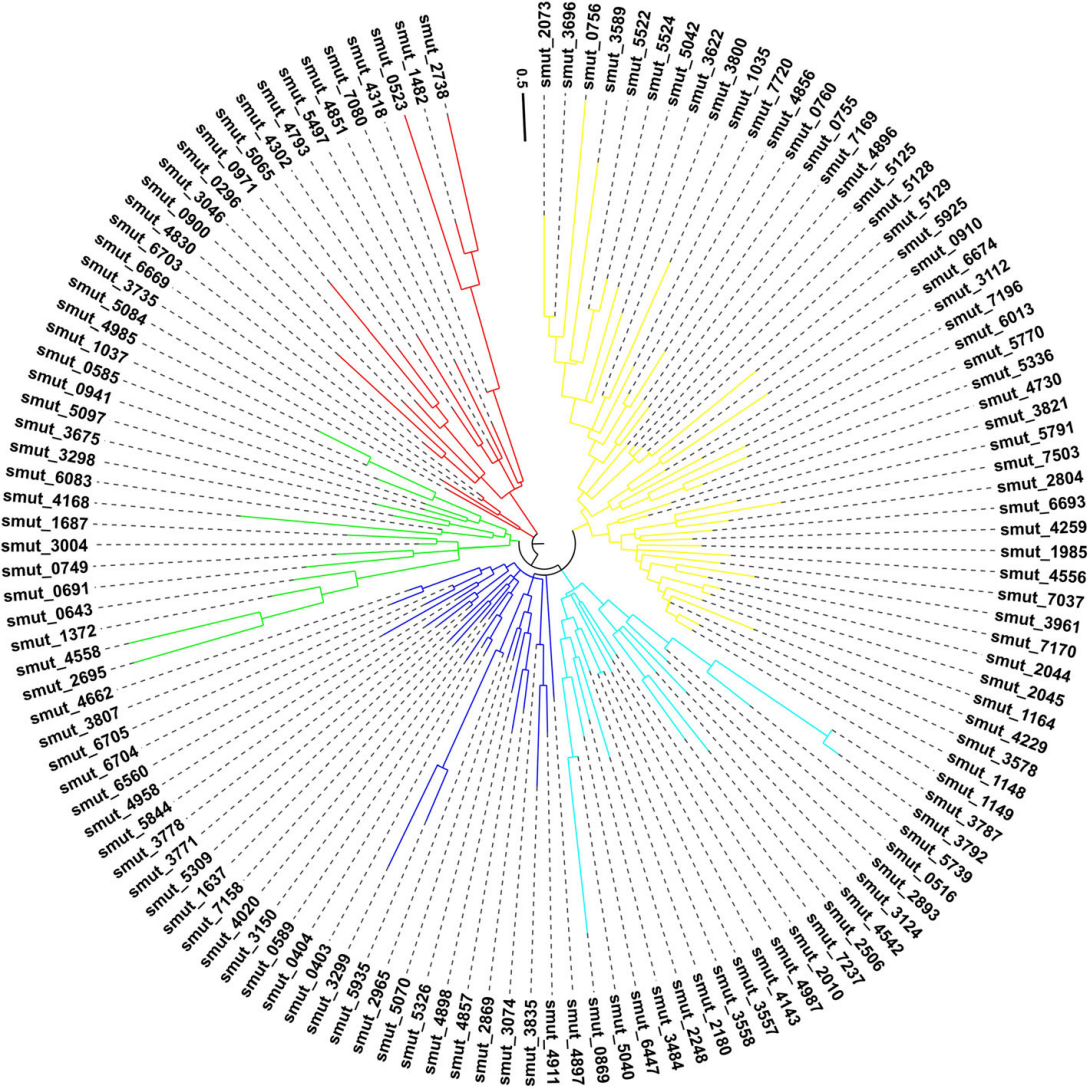
The phylogeny of the 131 putative effectors in *T. horrida*

Effectors are often characterized by their small size and high cysteine conten [24]. There were 367 small secreted proteins (≤400aa), and these comprised 61.47% of the *T. horrida* secretome, less than 67.19% in *T. caries*, and 66.01% in *T. controversa*; but greater than 60.06% in *T. indica* and 59% in *T. walker* (Table 1; Additional file 1: Table S1). Of these, 131 small secreted proteins that were up-regulated at 8 h after inoculation were selected as putative effectors, according to our previous study (Fig. 2) [23].

### Series-clustered analysis of secreted proteins

**Fig. 3:**
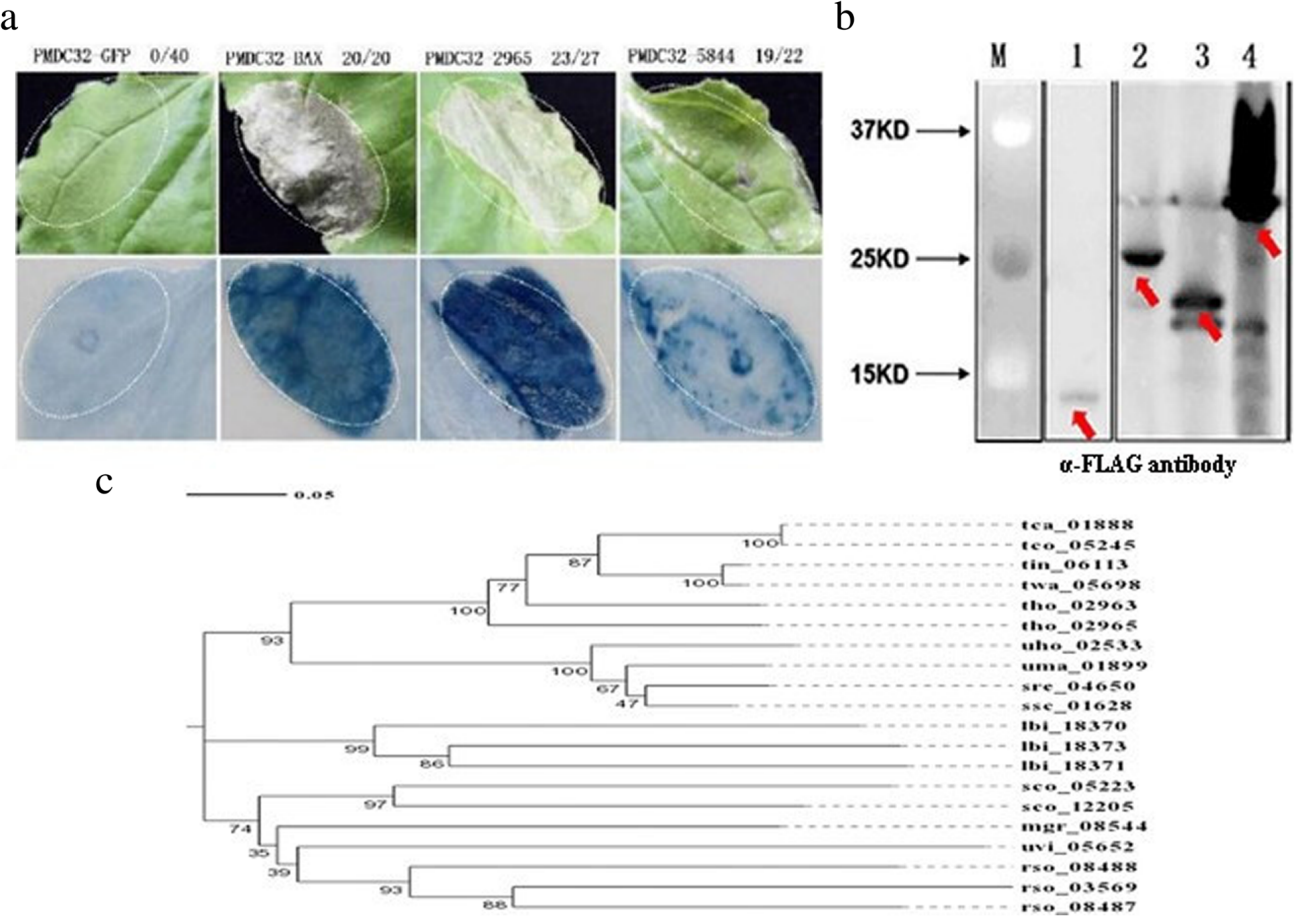
The putative effectors in *T. horrida* induce necrotic cell-death phenotypes in *Nicotiana benthamiana* and treated leaves were stained with Trypan blue. **a** The symptoms of *N. benthamiana* leaves after inoculation with smut_2965 and smut_5844. PMDC32-GFP means PMDC32 vector containing the green fluorescent protein (GFP) gene; the same labeling was used for PMDC32-BAX, PMDC32–2965, and PMDC32–5844. Numbers, e.g., 23/27, indicate that 23 of 27 infiltrated leaves exhibited cell death or mottling phenotypes. Agrobacterium carrying green fluorescent protein (GFP) served as a negative control. Agrobacterium carrying the BAX vector served as a positive control. Representative photos were taken four days after infiltration. **b** The expressed proteins were detected in the leaves of *N. benthamiana* by western blot experiments. M: marker; B1–4: smut_2965 (13.86 kDa), smut_5844 (24.84 kDa), BAX (21 kDa), and GFP (27 kDa). **c** The phylogeny of smut_2965 and 12 fungi homology secreted proteins. The phylogeny was constructed using Mega v7.0.26. tca denotes *Tilletia caries*, tco denotes *Tilletia controversa*, tin denotes *Tilletia indica*, twa denotes *Tilletia walkeri*, tho denotes *Tilletia horrida*, uho denotes *Ustilago hordei*, uma denotes *Ustilago maydis*, src denotes *Sporisorium reilianum*, ssc denotes *Sporisorium scitamineum*, lbi denotes *Laccaria bicolor*, sco denotes *Schizophyllum commune*, mgr denotes *Magnaporthe grisea*, uvi denotes *Ustilaginoidea virens*, and rso denotes *Rhizoctonia solani*

**Fig. 4:**
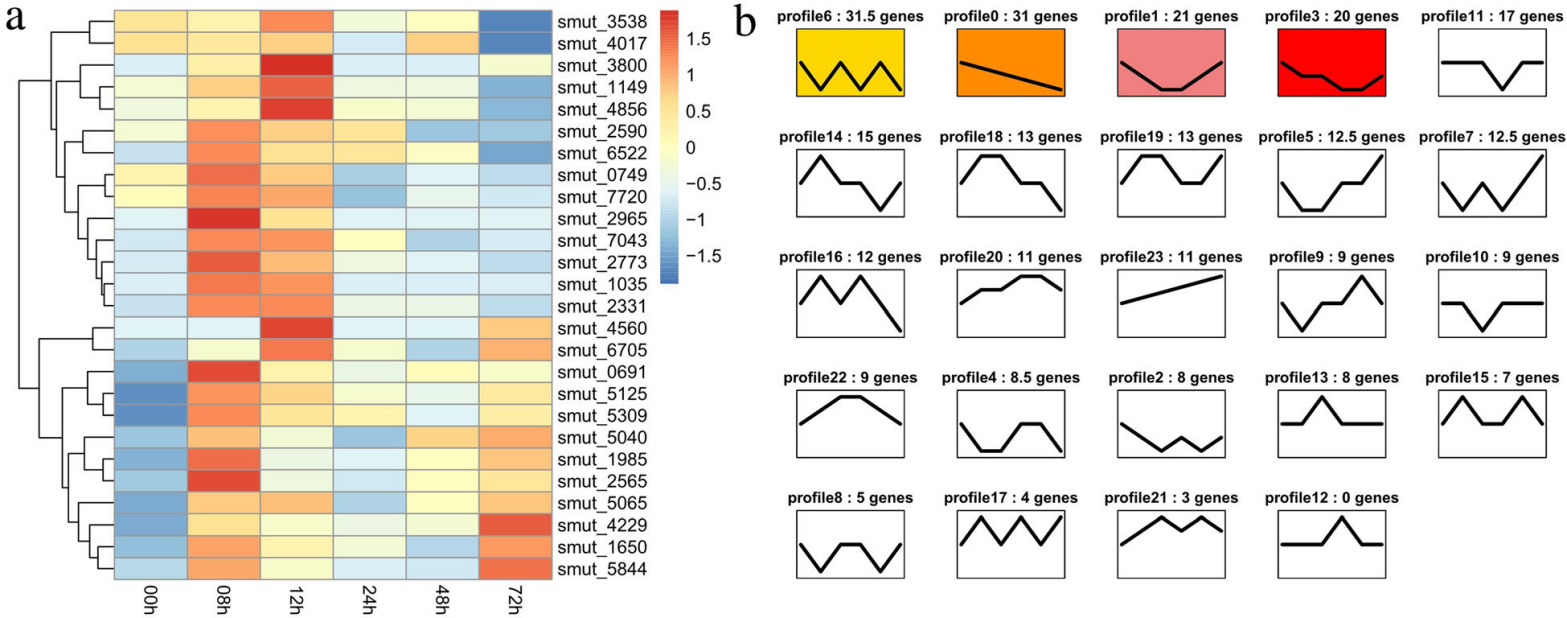
Trend analysis of putative effector genes. **a** The expression of 12 genes in profile 18 (smut_5844 was included) and 14 genes in profile 19 (smut_2965 was included). **b** The 291 differentially expressed genes that encoded secreted proteins were clustered in 23 profiles (no genes were found in profile 12). In the analysis, smut_3800 was clustered in profile 18

In our previous report, among 36 randomly selected predicted effectors (Additional file 2: Table S2), two effector genes, smut_2965 and smut_5844, were shown to trigger cell death in *N. benthamiana* leaves. However, a negative control of the green fluorescent protein (GFP) construct did not trigger the cell death phenotype (Fig. 3a). Western blot experiments showed that these proteins were expressed in the infiltrated leaves of *N. benthamiana* (Fig. 3b). In addition, we found several homolog genes of smut_2965 in five smut species (Fig. 3c). However, the homolog genes of smut_5844 were not found; we therefore predicted that it is a novel effector in *T. horrida*. To further select high confidence candidate effectors from among these genes that encode secreted proteins, expression trend clustering analysis of 291 differentially expressed genes (*P* ≤ 0.05 and |log_2_ fold change| ≥ 1) that encoded secreted proteins was performed at different infection time points, based on our previous transcriptome data [23]. This approach categorized the genes into 23 clustered profiles and enabled the selection of clusters with different sets of characteristics that may relate to different functions (Fig. 4). For example, profile 23 contained 11 genes, whose expression was up-regulated at five time points. These sustained up-regulated genes appeared to have important biological significance during infection. The clusters in profile 18, in which the expression pattern was similar to that of smut_2965; and the expression pattern of profile 19 that contained 13 putative effector genes and was similar to that of smut_5844, indicated that these genes may be involved in processes that occurred at the same stage of infection with smut_2965 and smut_5844 (Fig. 4a, b).

### Functional validation of predicted SPs of putative effectors

**Fig. 5:**
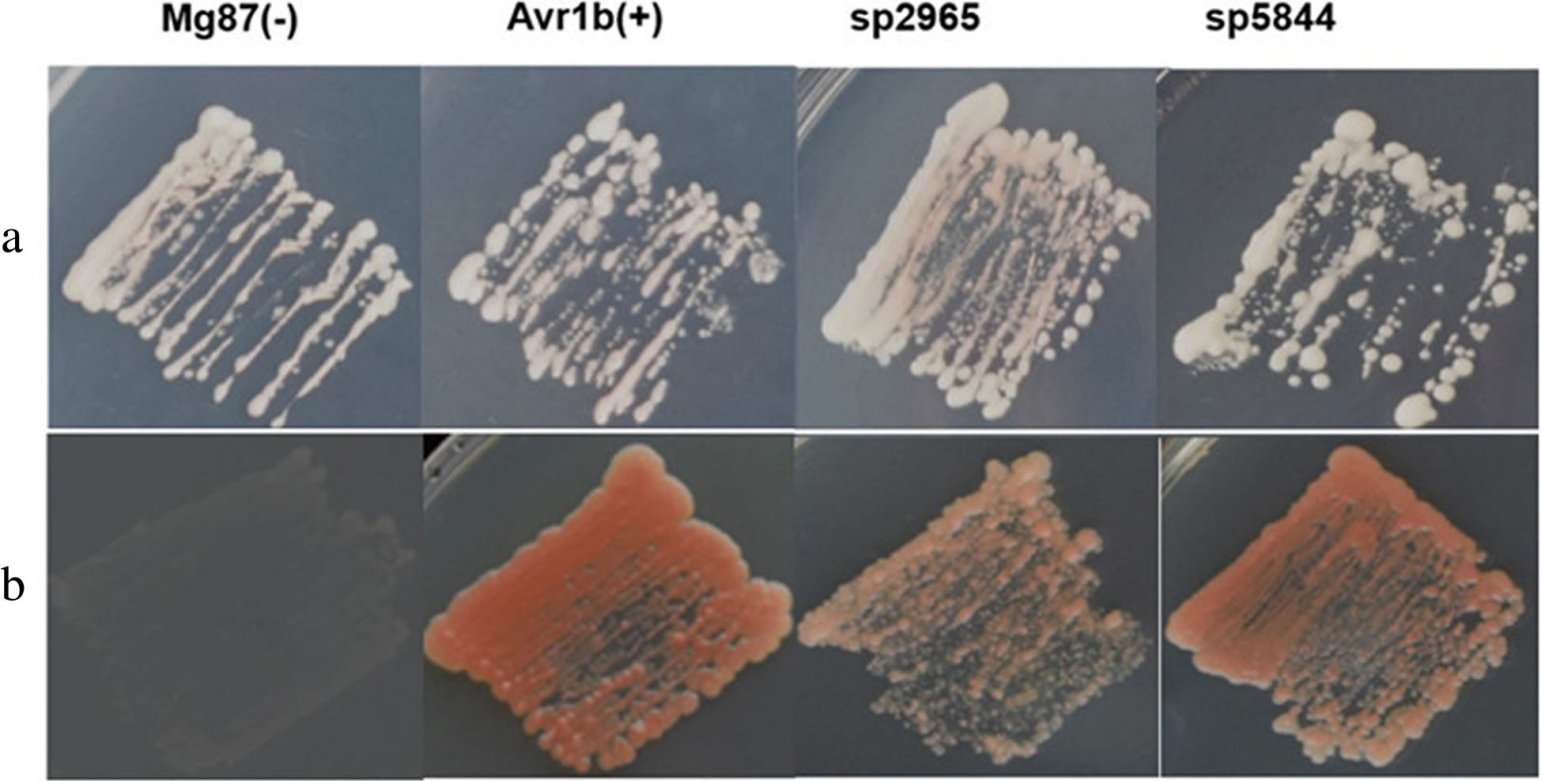
Functional validation of signal peptides (SPs) of putative *T. horrida* effectors using a yeast invertase secretion assay. The SPs predicted two *T. horrida* putative effectors. All transformed yeast strains YTK12 were able to grow on YPRAA media with raffinose as the sole carbon source (1% yeast extract, 2% peptone, 2% raffinose, and 2 μg of antimycin A per liter). The N-terminal sequences of *Phytophthora sojae* Avr1b and *Magnaporthe oryzae* Mg87 were used as positive and negative controls, respectively. The untransformed YTK12 did not grow on either CMD-W (0.67% yeast N base without amino acids, 0.075% tryptophan dropout supplement, 2% sucrose, 0.1% glucose, and 2% agar) or YPRAA media. Yeast growth on CMD-W media was equally viable among the transformed strains. Row **a**: CMD-W media; row **b**: YPRAA media; Mg87 (−): negative controls Mg87 SPs; Avr1b (+):positive controls Avr1b SPs; sp2965: SPs of smut_2965; sp5844: SPs of smut_5844

We used a yeast secretion system to experimentally identify the ability of predicted SPs of the smut_2965 (guanyl-specific ribonuclease, gsr1) and smut_5844 (unannotated, uan2) effector genes in *T. horrida* according to a method used in previous studies [25]. The predicted SP nucleotide sequence of gsr1 and uan2 were each fused in frame with the truncated pSUC2 gene that encodes invertase lacking its own SP. The constructed vectors were transformed into yeast strain YTK12, which is deficient in invertase secretion. Here, the invertase with functional SPs could degrade raffinose into simple sugars. The sugars therefore supplied a carbon source to YTK12 to enable it to grow on the medium with raffinose [26, 27]. The results showed that the predicted SPs of two putative effectors and a positive control (the secretion signal of *Phytophthora sojae* Avr1b) led to the secretion of invertase. These were grown on YPRAA medium with raffinose (Fig. 5). As a negative control, the N-terminus of Mg87 in *M. oryzae* [28] did not grow on YPRAA medium (Fig. 5). These results indicate that the predicted SPs of the two putative *T. horrida* effectors were functional secreted proteins.

### SPs are required for putative *T. horrida* effector uan2 to trigger plant cell death

**Fig. 6:**
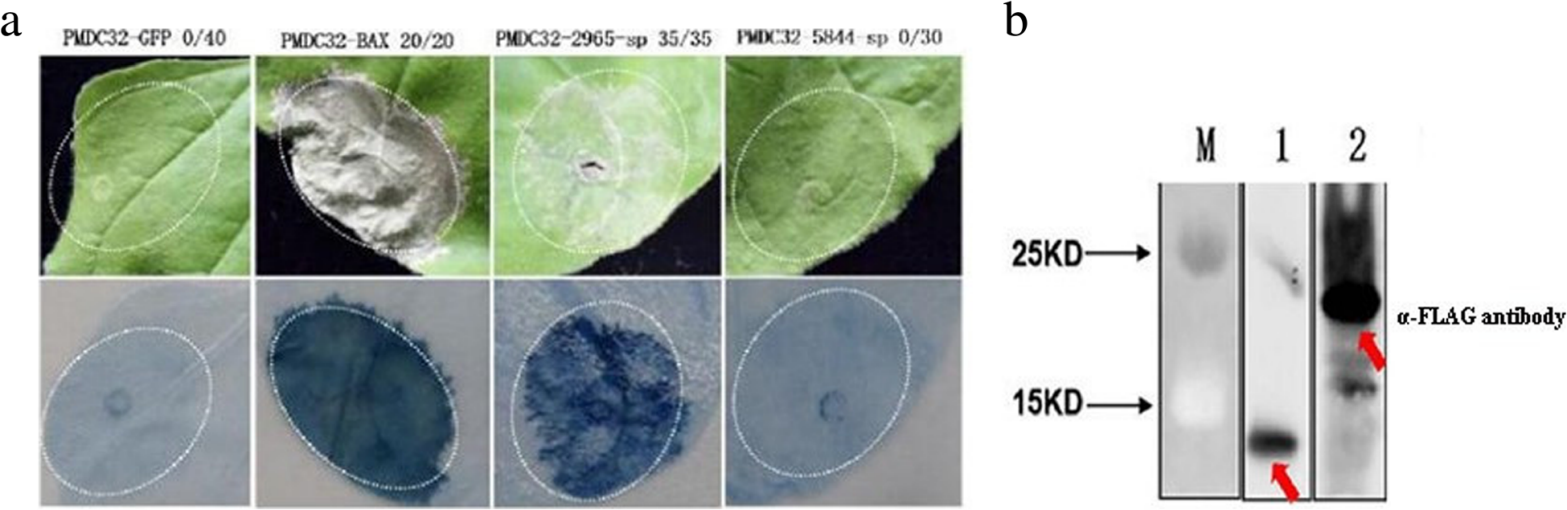
**a** The ability of truncated *T. horrida* secreted proteins lacking signal peptides (SPs) to induced necrotic cell-death. PMDC32–2965-sp denotes smut_2965 without SPs, PMDC32–5844-sp denote smut_5844 without SPs. Numbers, e.g., 20/20, indicate that 20 of 20 infiltrated leaves exhibited cell death or mottling phenotypes. **b** Expressed proteins were detected in the leaves of *N. benthamiana* following western blot experiments. M: marker; b1–2: smut_2965-sp (12.9 kDa) and smut_5844-sp (24 kDa)

According to previous reports, the SPs of many *M. oryzae* effectors are required for their ability to induce cell death in plants [29]. In this study, we identified whether two putative effectors in *T. horrida*, gsr1 and uan2, without SPs had the ability to induce cell death through transient expression assays in *N. benthamiana* leaves. The results showed that smut_5844-sp (uan2 lacking SPs) no longer caused a cell-death phenotype; however, smut_2965-sp (gsr1 lacking SPs) still had the ability to cause cell-death (Fig. 6a). Western blot experiments showed that proteins without SPs were expressed in the infiltrated leaves of *N. benthamiana* (Fig. 6b). Taken together, these results indicate that not all of the SPs of the tested secreted proteins in *T. horrida* are required to trigger cell death in *N. benthamiana*.

### The predicted RNase active site of gsr1 is essential for its cell death-inducing ability

**Fig. 7:**
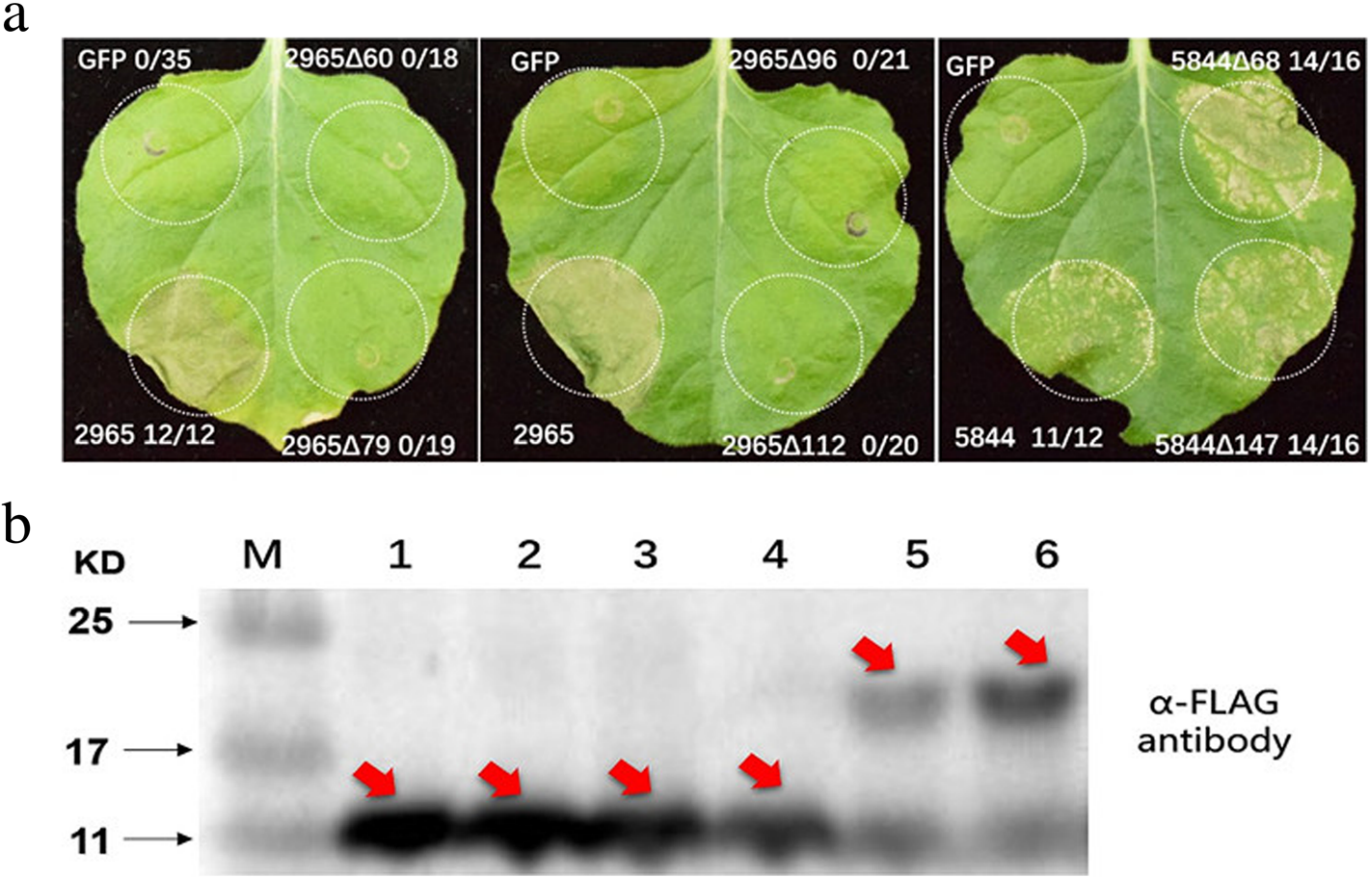
The predicted RNase active site of smut_2965 is required for its cell-death triggering ability. **a** The mutant proteins smut_2965–60, smut_2965–79, smut_2965–96, and smut_2965–112 lost the ability to induce cell-death, while smut_5844–68 and smut_5844–174 induced cell-death in *Nicotiana benthamiana* leaves. GFP refers to the PMDC32 vector containing the green fluorescent protein (GFP) gene, the same labeling is used for 2965, 5844, 2965∆60, 2965∆79, 2965∆96, 2965∆112, 5844∆68, and 5844∆147. Numbers, e.g., 0/35, indicate that 0 of 35 infiltrated leaves exhibiting cell-death or mottling phenotypes. **b** Protein expression of the smut_2965 and smut_5844 mutant proteins in the infiltrated leaves was detected by western blotting. M:marker; b1–6: smut_2965∆60 (13.74 kDa), smut_2965∆79 (13.74 kDa), smut_2965∆96 (13.74 kDa), smut_2965∆112 (13.74 kDa), smut_5844∆68 (24.72 kDa), and smut_5844∆147 (24.72 kDa)

It was predicted that gsr1 has four conserved RNase domains, and uan2 was predicted to contain two conserved RNase domains. To identify whether potential RNase activity was necessary for the ability of effector genes to induce cell death, we deleted the gsr1 predicted active site (Tyr-60, Glu-79, Arg-96, and His-112) and the uan2 predicted active site (Val-68, Leu-147) of the conserved RNase domain using a sequence synthesis technique. The full-length gsr1 and uan2, as well as the uan2 mutant proteins without predicted active sites caused cell death in *N. benthamiana* (Fig. 7a). However, the expression of gsr1 mutant proteins without predicted active sites in *N. benthamiana* did not induce cell death (Fig. 7a). Western blot analysis showed that these four gsr1 mutant proteins without predicted active sites were expressed in *N. benthamiana* leaves (Fig. 7b). These results indicate that the putative RNase active site of gsr1 is essential for its ability to induce plant cell death, but that this is not the case for uan2.

### Expression analysis of two putative effector genes during *T. horrida* infection of young panicles

**Fig. 8:**
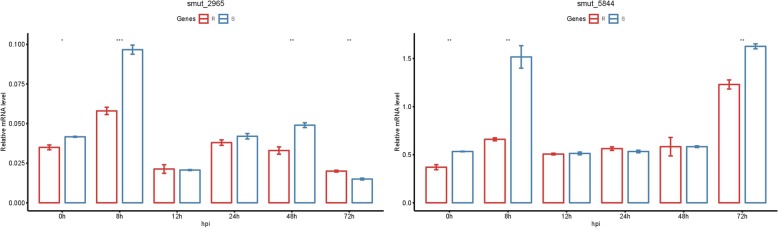
Expression profiles of two putative effector genes during *T. horrida* infection of the kernel smut–resistant and -susceptible rice male sterile lines. The *T. horrida*–inoculated panicles of the kernel smut–resistant cultivar Jiangcheng 3A (resistant rice male sterile line, R) and susceptible cultivar 9311A (susceptible rice male sterile line, S) were collected at 0, 8, 12, 24, 48, and 72 h post-inoculation for gene expression analyses using quantitative real time reverse transcription-polymerase chain reaction (qRT-PCR) assay. The UBQ gene was used to normalize the data, which are presented as normalized relative quantities scaled to the control. Error bars indicated the standard deviation of five independent replicates. For statistical analysis, we used analysis of variance with the R-package “ggpubr”, and significance was set at *P* < 0.05. *: *P* < 0.05, **: *P* < 0.01, ***: *P* < 0.001

In plant-pathogen interactions, the effector genes in filamentous pathogens are often transcriptionally induced [30]. To clarify the change in effector gene expression during *T. horrida* infection, the young panicles of rice male sterile lines Jiangcheng 3A (phenotype resistant to *T. horrida*), and 9311A (phenotype susceptible to *T. horrida*) were infected with the highly virulent isolate JY-521 [31]. The expression levels of these two secreted protein-encoding genes 8, 12, 24, 48, and 72 h after infection were identified by quantitative real time reverse transcription-polymerase chain reaction (qRT-PCR). These two effector genes were up-regulated 8 h after infection with *T. horrida* JY-521, and the level of gsr1 expression peaked at 8 h, while that of uan2 peaked at 72 h (Fig. 8). These results were consistent with the transcription data, and showed that both effector genes induced up-regulated expression during the early infection stage [23]. The findings suggest that these genes have important roles in host-fungal interactions.

### Identification of the *N. benthamiana* immune response induced by gsr1 and uan2

**Fig. 9:**
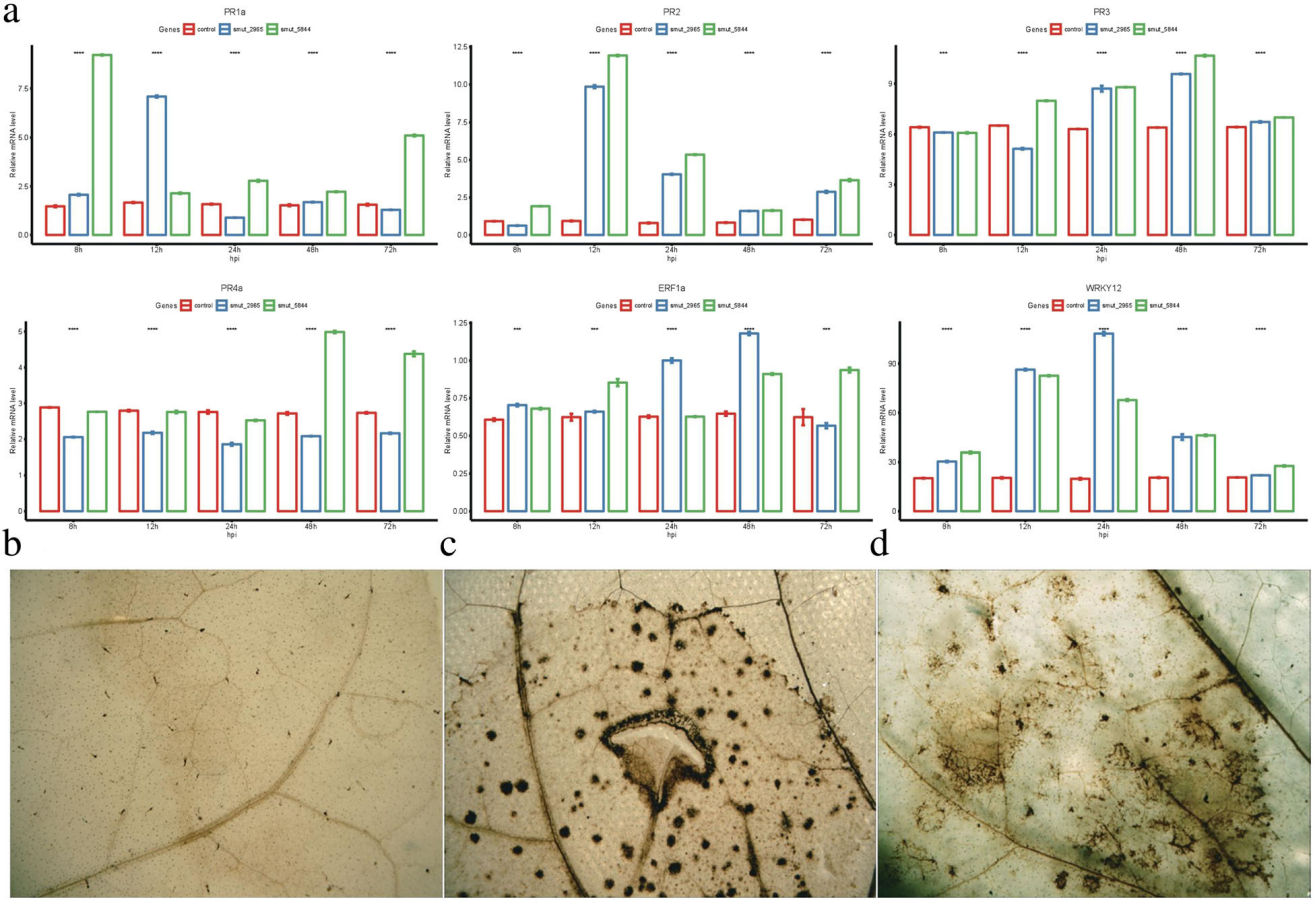
**a** Gene expression levels of related to plant immunity in *Nicotiana benthamiana* leaves transiently expressing two effector genes for 8, 12, 24, 48, and 72 h. *Nicotiana benthamiana* leaves were infiltrated with Agrobacterium tumefaciens GV3101 carrying either the PMDC32 empty (Control) or PMDC32-smut_5844 and smut_2965 vector. The plants were incubated in a plant growth room with 12 h/12 h, night/day photoperiods at 20–24 °C with 60% relative humidity. The leaves were harvested after 8, 12, 24, 48, and 72 h post-infiltration. Total RNA was extracted and reverse transcribed into cDNA. The qPCR was performed on the cDNA using specific primers for several genes related to activation of plant immunity. Actin gene was used to normalize the data, which are presented as normalized relative quantities scaled to control. Error bars indicated the standard deviation of 3 independent replicates. **b** *N. benthamiana* leaf that infected with agrobacterium carrying GFP was dyed used DAB. **c** *N. benthamiana* leaf that infected with agrobacterium carrying smut_2965 was dyed used DAB. **d** *N. benthamiana* leaf that infected with agrobacterium carrying smut_5844 was dyed used DAB. For statistical analysis, we used analysis of variance with the R-package “ggpubr”, and significance was set at P < 0.05. *: *P* < 0.05, **: *P* < 0.01, ***: *P* < 0.001, ****: *P* < 0.0001

We evaluated the expression levels of several genes related to the activation of the immune response in *N. benthamiana* using qRT-PCR. Among the selected pathogenicity related (PR) proteins, PR1a and PR2 were induced by uan2 at 8 and 24 h, respectively, compared with the control. By contrast, neither PR3 nor PR4a showed any significant transcriptional change compared with the control during the 72 h time course of the experiment. The transcription factor WRKY12 was also strongly induced at 12 h by these two effector genes (Fig. 9a). For gsr1, PR1a and PR2 were induced at 12 h (Fig. 9a). In addition, we investigated the ability of these two effector genes to induce the hydrogen peroxide (H_2_O_2_) mediated activation of *N. benthamiana* leaves using 3′-diaminobenzidine (DAB) dye [32]. The results showed that both effector genes could induce H_2_O_2_ activation at 72 h after infection compared with the control (Fig. 9b, c, d).

### Prediction of uan2-interacting proteins by a yeast two-hybrid analysis and transcriptome

**Fig. 10:**
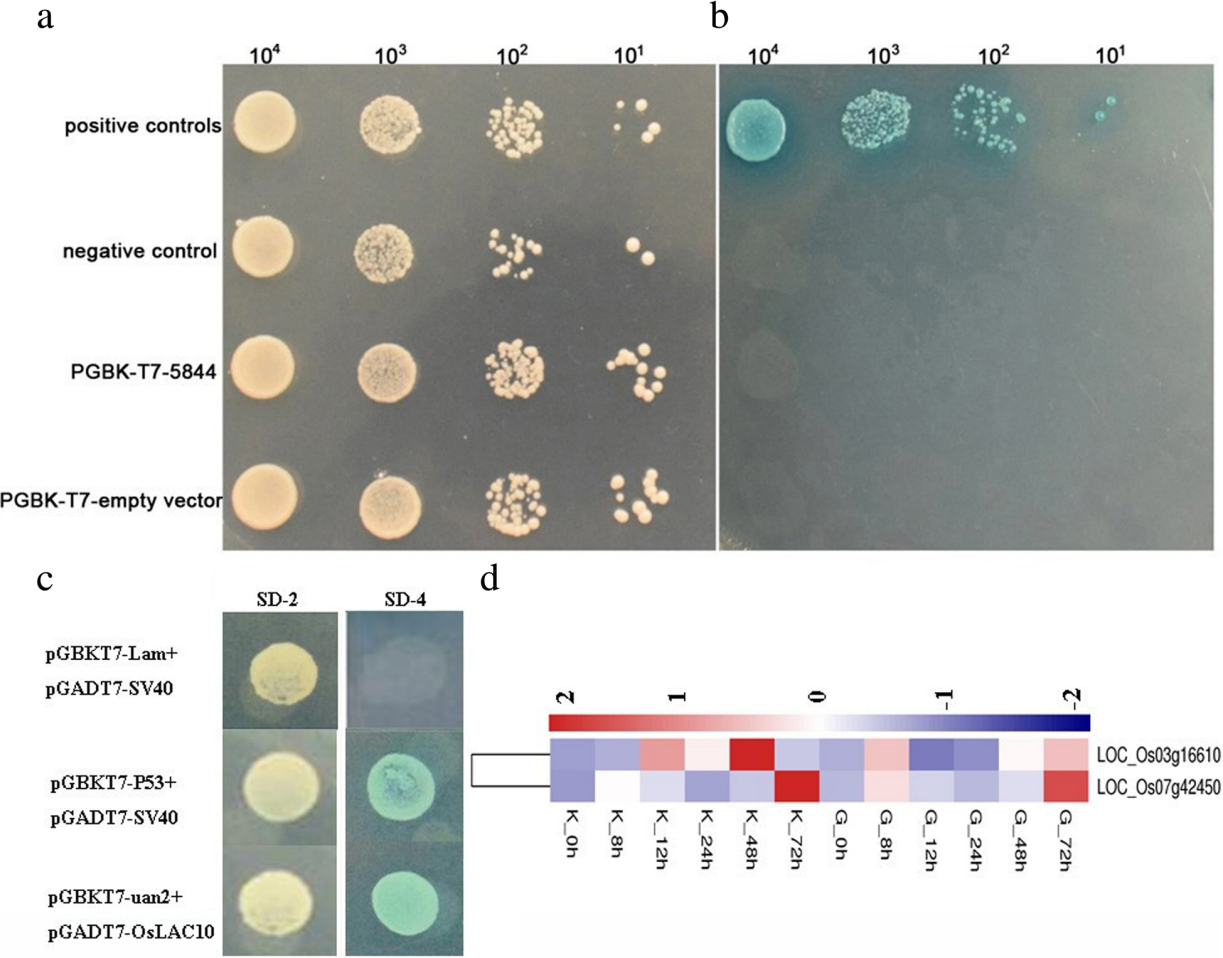
Yeast two-hybrid analysis of the smut_5844. **a-b** Autoactivation activity test of smut_5844 protein in yeast. a: SD-Trp/−Leu, b: SD-Trp/−Leu/−His/−Ade/X-α-gal. pAD-WT/pBD-WT was used as positive controls, and pAD-WT/pLaminC was used as a negative control. WT, Wild-type fragment C of lambda cI repressor (aa 132–236); Mut, E233K mutated fragment of lambda cl repressor (aa 132–236); Lamin C, human lamin C (aa 67–230). **c** Interaction between smut_5844 and OsLAC10 in a yeast two-hybrid system. P53 and SV40 were used as a pair of positive control. Lam and SV40 were used as a negative control. SD-2, SD-Trp-Leu; SD-4, SD-Trp-Leu-His-Ade. **d** The expression patterns of OsLAC10 and OsRbs5 in resistant and susceptible rice male sterile lines after *T. horrida* inoculation

The auto-activation test showed that positive controls (the pGBKT7–53 and pGADT7-T vector together) grew on both the SD-Trp/−Leu and SD-Trp/−Leu/−His/−Ade/Aba/X-α-gal plates; while a negative control (the pGBKT7-lam and pGADT7-T vectors together), the pGBKT7 empty vector, and the pGBKT7 vector with uan2 grew on the SD-Trp/−Leu plate, but not on the SD-Trp/−Leu/−His/−Ade/Aba/X-α-gal plate (Fig. 10a and b). These results indicate that uan2 does not exhibit auto-activation activity in yeast. To identify putative uan2-interacting proteins, the full sequence of uan2 was used as a bait to screen a rice cDNA library using yeast two-hybrid analysis and nine potential interactors were identified. The gene sequences that encoded these nine proteins are listed in Additional file 3: Table S3. Nucleotide sequence analysis further revealed that these nine proteins cDNA fragment encoding laccase precursor protein (LOC_Os03g16610), ribosomal protein S2 (LOC_Os07g42450), ARF-GTPase-activating protein (OsAGAP), chlororespiratory protein (CRR6), chaperone protein dnaJ (LOC_Os05g26926), ML domain protein (LOC_Os07g06590), retrotransposon protein (LOC_Os11g46020), OsFBX167 - F-box domain containing protein (LOC_Os05g30920), and endo-beta-N-acetylglucosaminidase (LOC_Os10g33350), respectively. However, only two genes LOC_Os03g16610 (OsLAC10) and LOC_Os07g42450 (OsRbs5) showed up-regulated expression after *T. horrida* inoculation according to the transcriptome data from our earlier report (Fig. 10d) [31]. In addition, laccase is associated with lignification and plays an important role in iron metabolism [33–36]. Hood et al. reported that the expression of a fungal laccase gene in transgenic maize is associated with kernel browning and limited germination [37]. These results further indicate that uan2 could interact with OsLAC10. To determine the interaction between uan2 and its potential partner, OsLAC10, a yeast two-hybrid assay, was performed via co-transformation of pGBKT7-uan2 and pGADT7-OsLAC10 in the Y_2_H Gold yeast strain. Yeast cells harboring both uan2 and OsLAC10 grew vigorously on both SD/Leu-Trp media and SD-Trp/−Leu/−His/−Ade/Aba/X-α-gal media (Fig. 10c).

## Discussion

Plant pathogen fungal effector proteins have important roles in host-pathogen interactions [38]. In the *T. horrida* genome, there are 597 genes encoding secreted proteins, of which 131 are predicted to be candidate effectors [23]. Transcriptome analyses showed that many secreted protein genes in *T. horrida* were up-regulated during early infection [23]. To clarify the function of fungal effectors, *N. benthamiana* has been extensively studied as a model system using agroinfiltration [39]. Several effectors that induce non-host cell death have been identified in *M. oryzae* by transient expression assays in *N. benthamiana* using agroinfiltration [40]. Similar studies have been reported in *P. sojae* [41]. In this study, only 36 candidates were randomly selected for a functional assay; there two putative effectors in *T. horrida* were demonstrated to trigger cell death phenotypes in *N. benthamiana*. Further experiments showed that these two putative effectors were up-regulated during the early stages of *T. horrida* infection (Fig. 8), which is a common feature of these two effector proteins. In addition, transcriptome data in our study may enable the candidate effectors to be investigated, and provide useful information for the further analysis of fungus–plant interactions. Future studies should aim to identify additional effectors in *T. horrida*.

In our study, we found that gsr1 genes in *T. horrida* did not require an SP to trigger cell death in *N. benthamiana*. Interestingly, many oomycete RxLR effectors, such as AVR3a KI, ATR1 NdWsB, and ATR13, when directly expressed in plant, did not require an SP to trigger a hypersensitive response and were, thus, recognized inside the plant cytoplasm [42]. The requirement of an SP for secreted proteins to induce cell death in plants indicates that these proteins might function in the extracellular space. The effector protein VmE02 secreted by the necrotrophic fungus *Valsa mali* could induce cell death with or without an SP, and has been identified as a secreted apoplastic fungal protein [43]. This finding is similar to that for effector SsCP1, an extracellular protein secreted by *Sclerotinia sclerotiorum*, which also triggers cell death without an SP [44]. The effectors AVR3a^KI^, ATR13, and ATR1^NdWsB^ have been found in plant cytoplasm, where SPs were not required for the triggering of cell death [45]. In addition, uan2 is a small cysteine-rich protein and SP was required for its ability of induced cell death. It is possible that the cysteine residues of uan2 form multiple disulfide bonds to stabilize theier tertiary structure. This stabilization would, in turn, protect uan2 against degradation by apoplastic plant proteases, as has been shown for other secreted apoplastic fungal proteins [46]. These results indicate the importance of the extracellular space for the function of uan2. Therefore, we speculate that uan2 may be an apoplastic effector.

We also identified the effector gene gsr1 as a fungus-specific RNase-like protein that possesses the ability to trigger cell death in *N. benthamiana*. Our results indicate that RNase active sites were necessary for the ability to trigger cell death. In the transient assay, necrosis was abrogated in the mutant lacking this domain. This was also observed to be the case for the effector protein UV_1423 secreted by *U. virens*; RNase active sites were essential to its cell death–inducing activity [22]. BEC1011 and BEC1054 are secreted RNase like effectors in the powdery mildew pathogen *Blumeria graminis* f. sp. *Hordei*, which play an important role in *B. graminis* infection; however, the contribution of BEC1011 and BEC1054 to *B. graminis* infection may not involve RNase activity, with a similar situation for uan2 [47]. Furthermore, SPs were required for uan2 to induce cell death in *N. benthamiana*. A similar phenomenon has been found in *U. virens* effector proteins [22]. The eight full-length effectors in *U. virens* are able to trigger cell-death in leaf of *N. benthamiana*; however, proteins without SPs lack this ability [22]. The effector protein MoCDIP1 in *M. oryzae*, also requires an SP to trigger cell death [48]. This suggests that different effectors in same pathogenic fungi may function in the host-pathogen interaction via different mechanisms.

We determined the ability of these two effector genes to trigger the activation of the *N. benthamiana* immune response by confirming the expression of pathogenesis-related genes and the induction of H_2_O_2_ mediated activation. Gsr1 and uan2 could induce PR1a and PR2 at different inoculation times compared with controls. The induction of PR1a and PR2 was related to the activity of salicylic acid, which triggers the response to biotrophic pathogens [49]. As *T. horrida* is a biotrophic pathogen, our results indicate that the gsr1 and uan2 proteins may act as biotrophic effectors that trigger a biotrophic-specific response in plant cells. The transcription regulator gene WRKY12, which is involved in plant cell-death and known to participate in the defense against soft rot disease in both Chinese cabbage and Arabidopsis [50], was also triggered in *N. benthamiana* leaves following the transient expression of gsr1 and uan2. The induction of WRKY12 showed that the symptoms observed in *N. benthamiana* leaves are associated with plant cell death. Hydrogen peroxide has an important role in plant disease-resistance [51, 52]. In our study, the activation of H_2_O_2_ was also triggered by gsr1 and uan2 in *N. benthamiana* leaves. In conclusion, the activation of the *N. benthamiana* immune system was induced by these two effector genes.

Plant pathogen effectors evolved rapidly, as shown by plant resistant genes, resulting in a diverse range of effector proteins involved in host evasion [53]. For the *T. horrida* genome, 131 putative effectors were grouped into five clusters, demonstrating that marked sequence diversity exists among these putative effectors. On the other hand, a degree of conservation was observed in effectors among the related fungal species. For example, we found several homolog genes of smut_2965 in five smut species. We identified two candidate effectors in *T. horrida* that induced non-host cell-death or a defense response. However, the specific molecular mechanisms by which these proteins exert their effects rice–*T. horrida* interaction require further study.

## Conclusions

We investigated the cell death–inducing ability of 131 putative effectors in *T. horrida* through transient expression assays. Two putative effectors were found to induce cell-death and defense machinery expression in *N. benthamiana*. The predicted SP is required for the cell death–inducing activity of uan2, and the predicted RNase active site of gsr1 is essential for its cell death-inducing ability. Our results provide a useful foundation for understanding the mechanisms underlying rice-*T. horrida* interaction.

## Methods

### Strains, plant materials, and growth conditions

Two rice cultivars that displayed marked differences in their susceptibility to infection by *T. horrida*, the resistant rice male sterile line Jiangcheng 3A and the susceptible rice male sterile line 9311A [31], were used in this study. They were provided by the Department of Rice Research Institute of Sichuan Agricultural University. The *T. horrida* strain JY-521 was cultured in PSA medium (potato 200 g, sucrose 20 g, agar 15 g, and distilled water 1000 ml) at 28 °C. Constructed expression plasmids were transferred to *Agrobacterium tumefaciens* GV3101, which was cultured in LB medium (yeast extract 5 g, 1% tryptone 10 g, NaCl 10 g, and distilled water 1000 ml). The yeast strain YTK12 was grown on YPDA medium (yeast extract 10 g, peptone 20 g, glucose 20 g, agar 20 g, adenine hemisulfate 0.03 g, and distilled water 1000 ml). Antibiotics and the concentrations used were as follows: kanamycin 50 μg _**˙**_ ml^− 1^, ampicillin 100 μg _**˙**_ ml^− 1^, and rifampin 25 μg _**˙**_ ml^− 1^. *N. benthamiana* plants were used for transient gene expression, and were grown under a 14 h day and 10 h night cycle at 23–25 °C with 60% relative humidity.

### Homology analysis of secreted proteins

The coding DNA sequences of predicted secreted proteins in nine smut fungal genomes were obtained and compared using BLASTP 2.6.0+ with an evalue ≤1e-7 [54]. Proteins passing this filter were clustered into families though the Markov clustering algorithm in OrthoMCL v1.4 [54, 55] with the options “–mode 3” -I as 2.0, and single-copy gene families of secreted proteins in nine smut fungal genomes were obtained. The protein sequence of each single-copy family was aligned by MUSCLE v3.8.31.

### Analysis of the expression levels of the candidate small secreted protein encoding genes

Transcriptome data were provided in our previous report [23]. A total of 291 differentially expressed genes in the *T. horrida* secreted protein were classified into 23 clustered profiles based on trends observed in gene expression using the Short Time-series Expression Miner software [56]. Clustered profiles with *P* ≤ 0.05 were considered to be statistically significant.

### RNA isolation and plasmid construction of *T. horrida* putative effector genes

Total RNA was extracted from *T. horrida* using a Fungal RNA kit (Omega, Biel, Switzerland), and cDNA was synthesized using a Transcriptor First strand cDNA synthesis kit (Roche, Basel, Switzerland). Secreted proteins and putative effectors in *T. horrida* were predicted according to a previous study [57]. The full-length secreted protein-encoding genes were amplified with Trans Start FastPfu Fly DNA Polymerase (TransGen Biotech, Beijing, China). All restriction enzymes and ClonExpress enzymes were used following the manufacturer’s instructions (Vazyme Biotech, Nanjing, China). Primers for these assays were designed using CE Design v1.03, based on our predicted gene sequences and included a BamHI site and a StuI site. The primer sequences are listed in Additional file 4: Table S4. The obtained cDNA of target genes were gel-purified with a gel purification kit (Omega) and cloned into the PMDC32 expression vector.

### Prediction and function validation of SPs

The SPs of two candidate effector genes were predicted using http://www.cbs.dtu.dk/services/SignalP/. Functional analysis of the predicted SP of two candidate effectors in *T. horrida* was performed using a yeast secretion assay according to a method described in a previous report [58]. The SP sequences of two predicted effector genes were cloned into pSUC2 vectors that had a truncated invertase gene, which lacked the start codon and SP sequence, and were then transformed into the yeast strain YTK12 using Frozen-EZ yeast transformation II kit (Zymo Research, Irvine, CA, USA). A 100 μl solution of transformants was inoculated into CMD-W medium (yeast N base without amino acids 6.7 g, tryptophan dropout supplement 0.75 g, sucrose 20 g, glucose 1 g, agar 15 g, and distilled water 1000 ml, pH = 5.8). A single colony of yeast was selected, and inoculated on CMD-W and YPRAA medium (yeast extract 10 g, peptone 20 g, raffinose 20 g, antimycin A 2 μg, agar l5 g, and distilled water 1000 ml, pH =5.8).

### Site deletion by sequence synthesis

The RNase active site deletion mutant sequences of putative effector genes smut_5844 and smut_2965 were obtained using BioXp™ 3200 System DNA synthesis instruments according to the manufacturer’s instructions [59], with analysis performed at Sangon Biotech, Shanghai, China. The mutated gene sequences were relegated into the PMDC32 vector. The mutated gene sequences are listed in Additional file 5: Table S5.

### *Agrobacterium tumefaciens*–mediated transient gene expression

We according to a method used in our previous studies to performed the *N. benthamiana* leaf transformation [23]. The infiltration experiment of each construct was totally for 20 leaves from different plants as repeated. The positive control using vector containing the BAX gene, and a vector containing the GFP gene inoculant was used as the negative control. Four days after infiltration, we will take note of the phenotype of cell death.

### Quantitative real time reverse transcription-polymerase chain resction (qRT-PCR) and 3′-Diaminobenzidine (DAB) and trypan blue dye

The resistant rice male sterile line Jiangcheng 3A and the rice male sterile line 9311A that is highly susceptible to *T. horrida* were infected with the *T. horrida* strain JY-521 [31]. The method of *T. horrida* inoculation according to our former reported [23]. Mycelia of *T. horrida* JY-521 were collected at five time points post inoculation (8, 12, 24, 48, and 72 h). Mycelia of *T. horrida* JY-521, with no infected rice kernel, served as the control (infection 0 h), and were immediately frozen in liquid nitrogen, and stored at − 80 °C. Total RNA of *T. horrida* JY-521 was isolated using the Omega Fungal RNA kit. The ubiquitin (UBQ) gene was used as an internal control for data normalization. *N. benthamiana* leaves were inoculated with *A. tumefaciens* containing either PMDC32-empty (Control) or PMDC32-smut_5844 and PMDC32-smut_2965 with three biological replicates each. Total RNA was extracted 8, 12, 24, 48, and 72 h post infiltration using the Omega Fungal RNA kit. Six genes that were related to the plant immune response (ERF1a, PR1a, PR2, PR3, PR4a, and WRKY12) were selected for qPCR. The actin gene was used as an internal control for data normalization. qRT-PCR was performed with a Bio-Rad CFX96 Real-Time PCR System (Bio-Rad, Foster City, CA, USA), according to the manufacturer’s instructions. The expression levels of genes were calculated using the 2^-∆∆Ct^ algorithm. The primers used for qRT-PCR are listed in Additional file 6: Table S6. After infection, DAB and trypan blue dyeing of *N. benthamiana* leaves were performed as described previously [60].

### Protein extraction and immunoblot analysis

*N. benthamiana* leaves inoculated with *A. tumefaciens* for 72–96 h were ground using liquid nitrogen. Protein extraction was performed using a one-step plant active protein extraction kit (Sangon Biotech, Shanghai, China) according to the instructions. The extracted proteins were separated using electrophoresis with 12% sodium dodecyl sulfate-polyacrylamide. To observe sample loading, the separated proteins were electrophoretically blotted on nitrocellulose membranes and stained using 0.1% Ponceau S. Five percent skimmed milk in TBS-T buffer (50 mM Tris-HCl, pH 7.5, 150 mM NaCl, 0.05% Tween 20) was used to block the membranes for 1 h at room temperature, followed by incubation for 1 h using anti-FLAG antibody solution (1:5, 000 dilution) at room temperature; the membranes were then washed using TBS-T buffer. Blots were incubated for 1 h with horseradish peroxidase-conjugated anti-mouse secondary antibody (1:5, 000 dilution in TBS-T) at room temperature. The immunoblots were incubated using the eECL western substrate and observed with X-films.

### Yeast two-hybrid analyses of protein–protein interactions

The auto-activation test and cDNA library screening were performed according to the instructions of the “HybriZAP-2.1 Two-Hybrid Libraries” system. The smut_5844 sequence was cloned to the pGBKT7 vector, and the pGBKT7 empty vector and pGBKT7 vector with smut_5844 were co-transformed into yeast strain Y_2_H and inoculated onto plates lacking tryptophan or leucine (SD-Trp/−Leu) for 2–3 d. Single colonies were selected and inoculated into fluid SD-Trp/−Leu medium for 2 d; Different concentrations of bacterial liquid were inoculated onto SD-Trp/−Leu and SD plates without tryptophan, leucine, histidine, and adenine, supplemented with 50 μg/ml Aureobasidin A (Aba) and 20 μg/ml X-α-gal (SD-Trp/−Leu/−His/−Ade/Aba/X-α-gal) for the auto-activation test. The pGBKT53 and pGADT7-T vectors served as positive controls, while the pGBKT7-lam with pGADT7-T vector served as a negative control. For cDNA library screening, the bait constructed (pGBKT7-smut_5844) and pGAD-T7-cDNA plasmid libraries were sequentially transformed into Y_2_H strains. The transformed yeast strains were then spread on SD-Trp/−Leu/−His/−Ade/Aba/X-α-gal plates and incubated at 28 °C for 2–4 d until colonies appeared. The blue colonies were then selected as potentially interacting candidates. The OsLAC10 coding region was cloned into pGADT7 based on the sequencing result. Yeast transformation was conducted following the Yeast Transformation Protocol (Clontech).

## Additional files

Additional file 1: **Table S1.** The predicted secreted proteins of nine smut fungus. (XLSX 67 kb)

Additional file 2: **Table S2.** The 36 randomly selected predicted effectors from 131 predicted effectors. (XLSX 10 kb)

Additional file 3: **Table S3.** The sequence of smut_2965, smut_5844, and potential interacting proteins with smut_5844. (XLSX 12 kb)

Additional file 4: **Table S4.** The primer sequence of two effector genes. (XLSX 9 kb)

Additional file 5: **Table S5.** The sequence of mutated genes. (XLSX 9 kb)

Additional file 6: **Table S6.** The primer sequence of qRT-PCR. (XLSX 8 kb)

## Data Availability

The datasets used and/or analyzed during the current study are available from the corresponding author on reasonable request.

## Abbreviations

GFP: Green fluorescent protein
H_2_O_2_: Hydrogen peroxide
PR: Pathogenicity related
qRT-PCR: Quantitative real time reverse transcription-polymerase chain reaction
RKS: Rice kernel smut
RT: Room temperature
SPs: Signal peptides
STEM: Short Time-series Expression Miner software
